# Fabrication of an AMC/MMT Fluorescence Composite for its Detection of Cr(VI) in Water

**DOI:** 10.3389/fchem.2018.00367

**Published:** 2018-08-21

**Authors:** Yanke Wei, Lefu Mei, Rui Li, Meng Liu, Guocheng Lv, Jianle Weng, Libing Liao, Zhaohui Li, Lin Lu

**Affiliations:** ^1^Beijing Key Laboratory of Materials Utilization of Nonmetallic Minerals and Solid Wastes, National Laboratory of Mineral Materials, School of Materials Science and Technology, China University of Geosciences, Beijing, China; ^2^State Grid Corporation of China, Beijing, China; ^3^Geosciences Department, University of Wisconsin—Parkside, Kenosha, WI, United States

**Keywords:** AMC/MMT composite, fluorescence, quenching, detection, hexavalent chromium

## Abstract

Hexavalent chromium species, Cr(VI), which can activate teratogenic processes, disturb DNA synthesis and induce mutagenic changes resulting in malignant tumors. The detection and quantification of Cr(VI) is very necessary. One of the rapid and simple methods for contaminant analysis is fluorescence detection using organic dye molecules. Its application is limited owing to concentration quenching due to aggregation of fluorescent molecules. In this study, we successfully intercalated 7-amino-4-methylcoumarin (AMC) into the interlayer space of montmorillonite (MMT), significantly inhibited fluorescence quenching. Due to enhanced fluorescence property, the composite was fabricated into a film with chitosan to detect Cr(VI) in water. Cr(VI) can be detected in aqueous solution by instruments excellent, ranging from 0.005 to 100 mM with a detection limit of 5 μM.

## Introduction

Due to the high toxicity of heavy metals and bioaccumulation in human body through the food chain, will bring human health and environmental great issue. Cr is one of the most serious contaminants among various heavy metals (Soewu et al., 2014; Wu et al., 2015a; Širić et al., 2017). While Cr(III) is an essential micronutrient, Cr(VI) is toxic (Weibel et al., 2016). Cr(VI) is mainly produced by electroplating, leather tanning, and textile dyeing (Dehghani et al., 2016; Omorogie et al., 2016). Cr(VI) is the most virulent form (Miretzky and Cirelli, 2010) of Cr, which is a highly toxic agent and act as carcinogens, mutagens, and teratogens in biological systems (Hlihor et al., 2017; Mullick et al., 2017). The carcinogenic and toxicity of Cr(VI) is based on its oxidation states like the most transition metals (Yusof and Malek, 2009; Kumar et al., 2017). An effective, simple and low-cost method for detecting Cr(VI) is needed (Babu and Gupta, 2008; Wang G. et al., 2017). The classic methods for detecting Cr(VI) include electrochemical method, atomic absorption method and liquid chromatography (Anthemidis et al., 2002; De Ruiter et al., 2008). These methods are credible. However, the processes are complicated. Due to its sensitivity, fluorescence spectroscopy has become one of the most increasingly used techniques (Wang et al., 2015; Li and Wei, 2017). The sensors made of organic or inorganic materials show great promise in detection, lighting, and other application (Rao et al., 2014).

Montmorillonite is a typical phyllosilicate of the smectite group of clay minerals (Ismadji et al., 2016; Xu et al., 2016). It is consists of two layers of silicon tetrahedron (T) and the middle layer of aluminum octahedron (O) (Bekri-Abbes and Srasra, 2016; Scholtzová et al., 2016; Wang W. et al., 2017). Because of the isomorphous replacement of Al^3+^ by Mg^2+^ and Fe^2+^ in the octahedral sites and Si^4+^ by Al^3+^ in the tetrahedral sites, TOT layers are negative electric charge, balanced by the cations such as Ca^2+^ and Na^+^ intercalate into the layers (Dominijanni and Manassero, 2012; Wu et al., 2016; Zhou et al., 2016). Luminescent materials of small organic molecules are easily modified and chemically purified to have wider emission spectrum coverage (Wu et al., 2015b; Tian et al., 2017). However, when they are applied in solid devices, stabilize and enhance their luminous intensity is crucial. Although several methods of assembling materials have been developed (Zhang et al., 2005), they often suffer from phase separation, agminated organic small molecules (Wei et al., 2016; De Sa et al., 2017). Clay minerals were rarely used as base materials for the organic-inorganic composite preparation for luminescence and fluorescence applications.

This research focused on the fabrication of a fluorescence material for Cr(VI) detection. To prevent quenching, the photoactive organic dye AMC was interposed into the interlining of MMT for maximal dye separation and minimum agminated (Yu et al., 2013, 2014). The AMC/MMT was fabricated in a film with chitosan. Quenching of fluorescence was used various solutions to evaluated. The response of AMC/MMT composites in Cr(VI) was excellent, ranging from 0.005 to 100 mM.

## Materials and methods

### Materials

**Figure 1:**
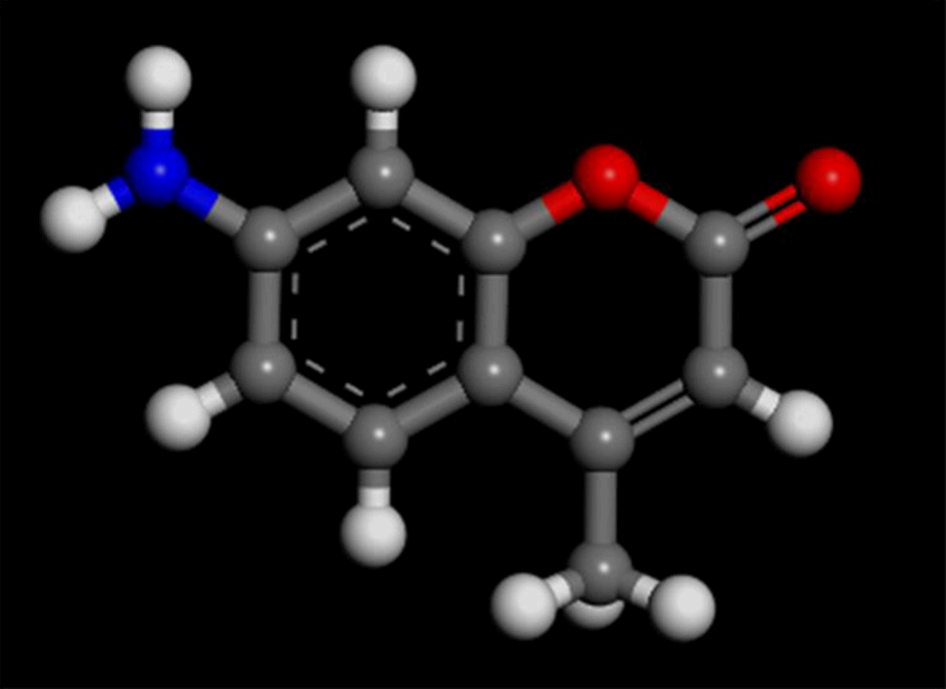
Molecular structure of AMC studied. The all strains, C, gray; N, blue; O, red; H, white.

The montmorillonite was bought from Ningcheng, Inner Mongolia, China. Its CEC (Cation Exchange Capacity) was 9.8 mmol_c_/10 g which used Na^+^ as the staple commutable cationan exchange. The specific external surface area was 78 m^2^/g. AMC was purchased from Aladdin. The molecular structure (Nowakowska et al., 2001) of AMC is presented in Figure 1. The potassium dichromate Cr(VI), chitosan, and other reagents were purchased from various manufactures that were all of analytical grade. Deionized water was used as a solvent.

### Preparation of AMC/MMT

The effect of initial AMC concentrations on a performance of florescence were tested, 50 mL the original concentrations of AMC in water for 20, 50, 100, 200, 500, and 800 mg/L were blended with 0.25 g of MMT in every 100 mL centrifugal tube after shaking at 200 rotations per minute at indoor temperature for 8 h, respectively. The admixtures were centrifuged at 7,500 rotations per minute for 2 min. Afterwards, removing the supernatant, then at 60°C were dried the residues and pulverized the residues to powder as raw materials. This type of product was used for the AMC/MMT.

### Preparation of the chitosan film (AMF) for Cr(VI) detection in water

The chitosan film was prepared as follows: 0.25 g chitosan, 8 mL 0.1 M NaOH solution, 0.1 g AMC/MMT powder and two drops of 1 g/mL Polyvinyl Alcohol (PVA) solution were appended. The admixture was whipped for 30 min and then poured into a mold and air dried.

The followed solutions at a concentration of 0.1 M were originally screened for quenching of fluorescence in AMF: Al^3+^, Ca^2+^, Ba^2+^, Cr(VI), CTAB, K^+^, Na^+^, Ni^3+^, Pb^2+^, Fe^3+^, Imidazole, C_2_H_5_OH. Afterwards, they were dropped onto the AMF. Then, the fluorescence degrees were measured using a fluorescence spectrophotometer (Hitachi, F4600).

In order to estimate the Cr(VI) range of response, different concentrations of Cr(VI) solution were dropped onto the AMF. The Cr(VI) of original concentrations were the ranged of 5 μM to 100 mM.

Pictures of the AMF after in contact with Cr(VI) solutions were already obvious demonstration quenching of fluorescence effect by Cr(VI) and then measure the florescence intensions of AMF by fluorescence spectrophotometer (Hitachi, F4600).

### Instrumental analyses

Powder X-Ray Diffraction (XRD) was performed by a Rigaku D/max-IIIa diffractometer (Tokyo, Japan) with a Ni-filtered Cu Ka radiation at 40 kV and 40 mA. Prototypes were scanned the range of 3° to 70° at 8° per minute with a pace of 0.01° to research the particular changes in d_001_ separation distance of MMT as a function of original AMC concentrations. The changing of peak position proved the intercalation of AMC into the MMT was succeeded. The gallery heights were deduced from the (001) reflection of the composites using the Bragg's equation.

Fourier Transform Infrared Spectroscopy (FTIR) spectra were acquired on a Perkin Elmer Spectrum 100 Spectrometer. The spectra were obtained from 4,000 to 500 cm^−1^ by adding 256 scans in the resolution ratio of 4 cm^−1^.

Photoluminescence emission (PL) spectrum was obtained on a fluorescence spectrophotometer (Hitachi, F4600) at the scope of 400–600 nm with a photomultiplier tube handled at 800 volt. A 150 watt xenon lamp was served as the pumping source, at an excitation wavelength of 340 nm (Zamojć et al., 2015). The emission and excitation slits size were 5 nm and scan speed was setting at 240 nm per min.

Molecular mimicry was performed at the standard module “Forcite” of Materials Studio 6.0 software to research the structure of AMC in the interval of MMT. The unit lattice parameters were setted at a = 1.55 nm, b = 1.79 nm, c = 1.25 nm, α = γ = 90°, and β = 99°. A suite of 2 × 2 × 1 supercells were constructed of the interlayer spacing setting at 1.63 nm. Every circulation consisted of 10^6^ steps, repeating three cycles.

## Results and disscussion

### The MMT was intercalated with AMC

**Figure 2:**
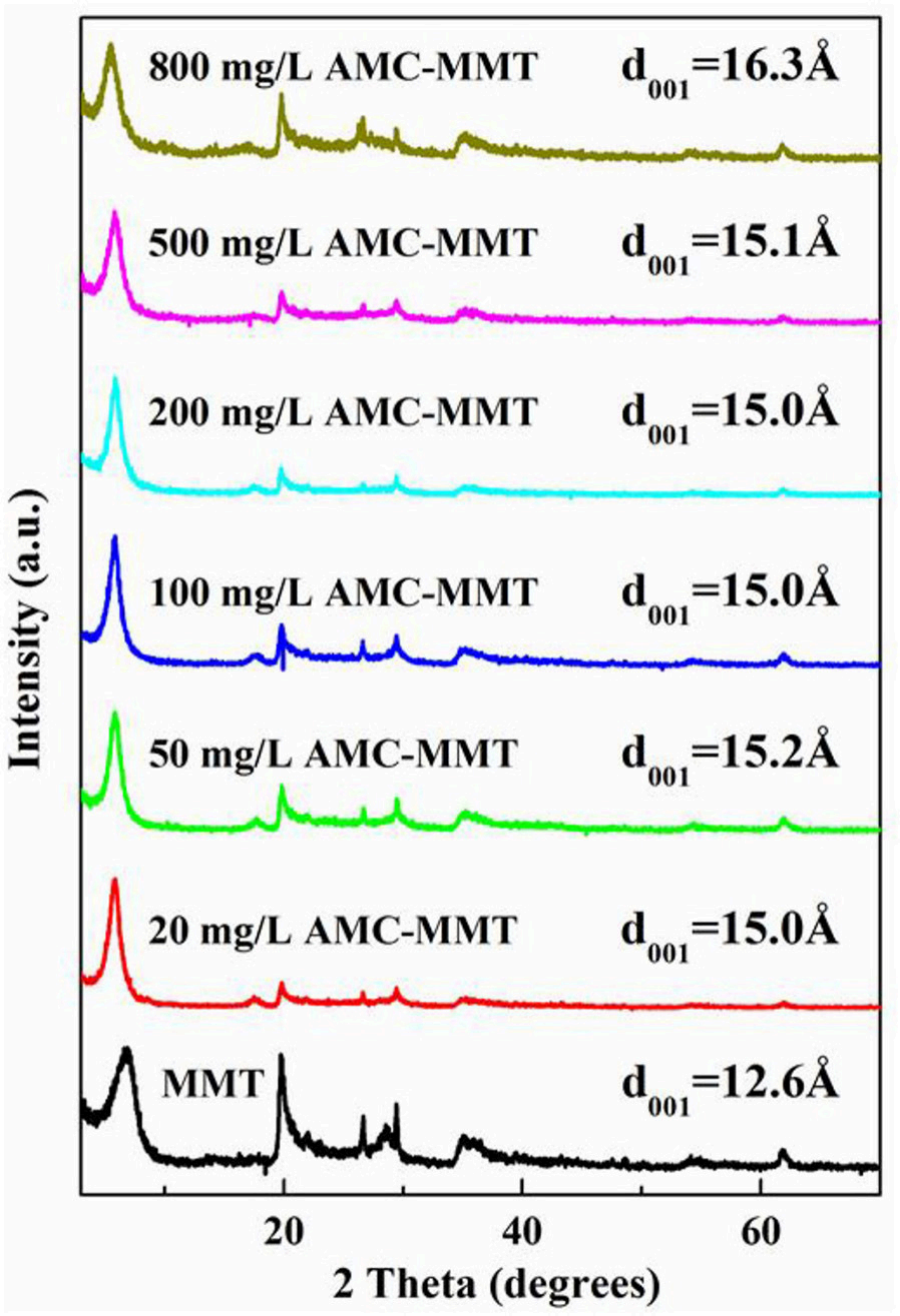
X-ray diffraction patterns of MMT was inserted with AMC in the interlayer, which was affected by initial AMC concentrations (mg/L).

The idea that interlayer cation was Na^+^ with a monolayer hydration was confirmed because the d_001_ value of pure MMT was 12.6 Å. The d_001_ spacing progressively increased from 12.6 to 16.3 Å with the initial AMC concentration increased (Figure 2), suggesting AMC molecules insert the portion between the interlayer space of MMT.

### FTIR spectra of AMC/MMT

**Figure 3:**
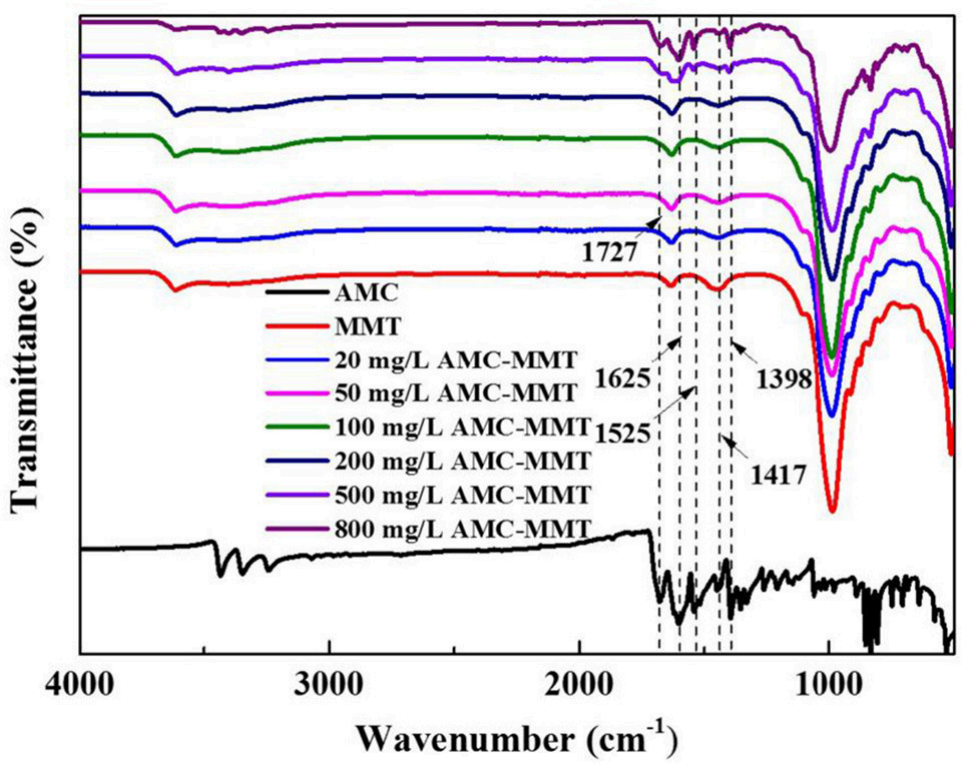
FTIR spectra of AMC, and MMT with different amounts of AMC adsorption.

FTIR spectra of MMT adsorbed with different amounts of AMC displayed both bands of raw MMT and AMC (Figure 3). The bands between 1,400 and 1,650 cm^−1^ were allocated to C-C stretching vibrations in aromatic and hetero aromatic compounds (Talbi et al., 1998; Krishnakumar and Xavier, 2005). Therefore, the bands observed at 1,727, 1,695, 1,625, 1,566, 1,525, 1,475, 1,446, 1,417, 1,398, 1,390 cm^−1^ in FTIR spectra were allocated to C-C stretching vibrations of AMC (Arivazhagana et al., 2010). The characteristic peaks of AMC appeared in FTIR spectra of AMC/MMT, as shown in the dotted line (Figure 3), indicating that AMC had been loaded onto MMT.

### Luminescence properties of AMC/MMT

**Figure 4:**
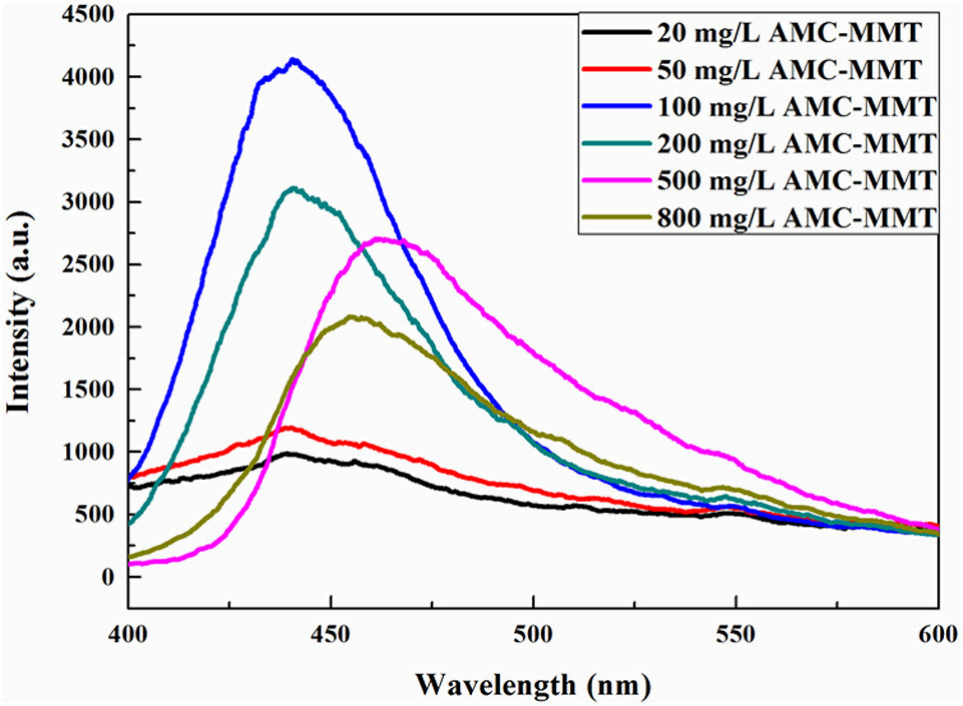
Fluorescence spectra of AMC/MMT was Influenced by initial concentrations of AMC.

AMC powder was hardly used as a luminescent material just because the lack of strong luminescence (Wang et al., 2012), causing by concentration quenching (Wu et al., 2015b). The luminous intensity increased significantly after the intercalation of AMC into the interlayer of MMT, which reason was AMC was uniformly dispersed on MMT, inhibiting its aggregation. The highest spectral intensity was found by AMC/MMT at 100 mg/L, which was an initial concentration (Figure 4).

### The fluorescent detection of Cr(VI) in aqueous phase by AMF

**Figure 5:**
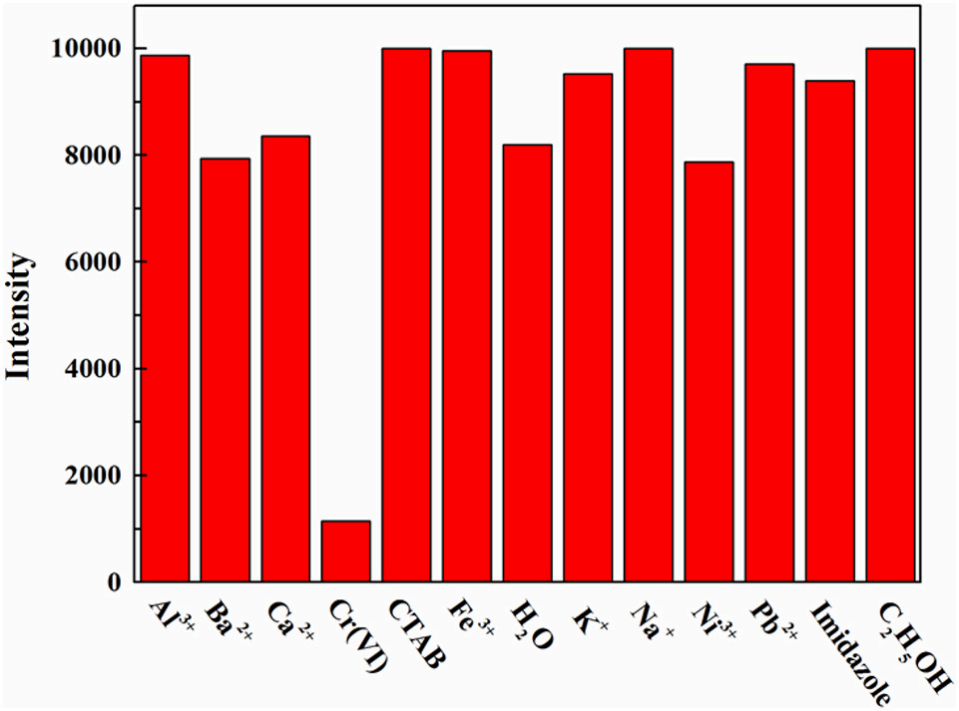
Fluorescence intensity of the AMF in response to a variety of aqueous solutions at a concentration of 0.1 M.

The AMF was screened for response to various solutes (Figure 5). The fluorescence response to Cr(VI) was very significant when the test solute with an initial concentration of 0.1 M, while others were not obvious, indicating the high selectivity of AMF for Cr(VI).

**Figure 6:**
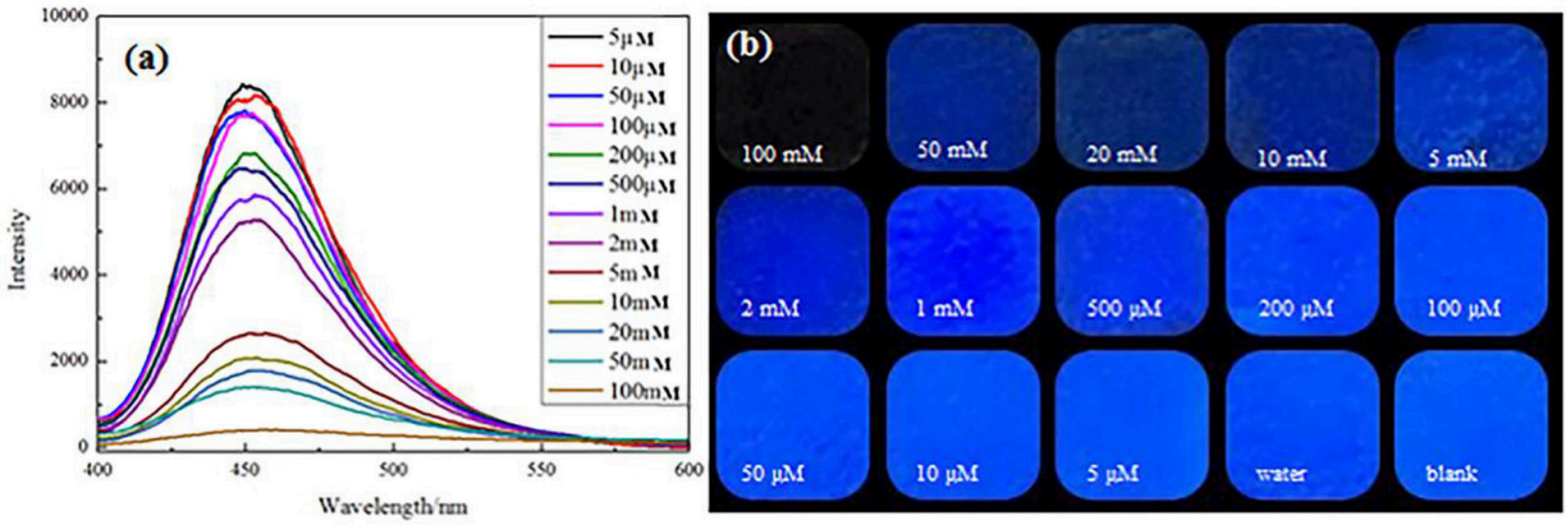
Fluorescence spectra of the AMF in the presence of multifarious concentrations of Cr(VI) **(a)**. Pictures of the AMF with disparate concentrations of Cr(VI) exposed UV lamp **(b)**.

The mechanism of photoluminescence (PL) quenching of AFM was further investigated when the aqueous solution contains Cr(VI). Most importantly, an apparent decrease in fluorescence intensity was observed while the Cr(VI) concentration was increasing over the whole concentration ranges from 0.005 to 100 mM (Figure 6a). A full PL quenching process can be observed visually at a Cr(VI) concentration of 1 mM. The images of AMF with addition of Cr(VI) that exposed ultraviolet light showed a consistent tendency (Figure 6b).

### Mechanism analysis

**Figure 7:**
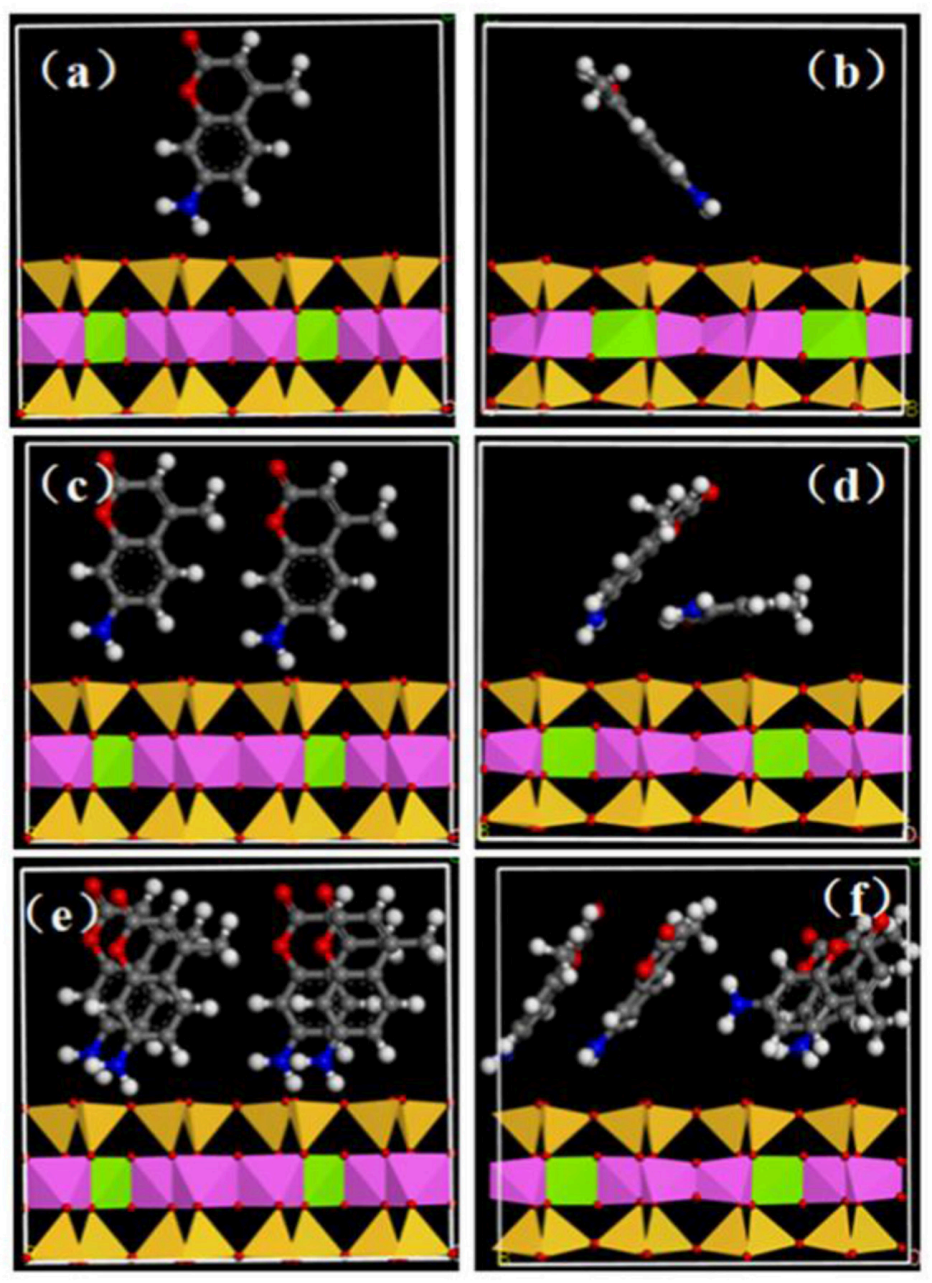
Dynamic simulation of molecular interposition AMC into MMT at a different angle on account of the electric density and AMC loading distinction. The initial states were represented by **a, c**, and **e**; and **b, d**, and **f** represent the states after simulation: **a** stands for low concentration, **c** stands for medium concentration and **e** stands for higher concentration. The all strains, Mg, green; Al, pink; Si, yellow; C, gray; N, blue; O, red; H, white.

Molecular mimicry was performed at the standard module “Forcite” of Materials Studio 6.0 software to research the structure of AMC between the interval of MMT. The interlayer spacing of MMT was directly influenced by the interlayer arrangement of AMC and the amount of AMC intercalation, this, on the other hand, would play an significant role in understanding the system structure and interaction forces (Krauss et al., 2011; Wang et al., 2011; Tournassat et al., 2016). The interlayer condition of AMC interpolation MMT with different quantities was qualitatively simulated, and the initial states were represented by a, c, and e; and b, d, and f represent the states after simulation (a stands for low concentration; c stands for medium concentration; and e stands for higher concentration; Figure 7). The concentration quenching could be effectively avoid by AMC intercalation MMT and it could also significantly improve luminous intensity, lifespan, and stability notably owing to the stronger interaction forces between AMC molecules and MMT sheet (Yan et al., 2010a,b; Liu et al., 2014).

## Conclusions

In this research, an organic dye (AMC) was resoundingly intercalated into the interlayer space of MMT, resulting in observably inhibition in quenching of fluorescence. The composite was fabricated into a film with chitosan to detect Cr(VI) in water with its enhanced fluorescent property. Cr(VI) can be detected in aqueous solution by instruments ranging from 0.005 to 100 mM with a detection limit of 5 μM.

## Author contributions

YW and LM conceived the project. LM, GL, and LibL designed and performed the experiments. RL, ML, JW, and LinL analyzed the data. YW, ZL, and LM wrote the manuscript.

### Conflict of interest statement

The authors declare that the research was conducted in the absence of any commercial or financial relationships that could be construed as a potential conflict of interest.
